# Clinical efficacy of a novel method of fertility-preserving adenomyomectomy in infertile women with diffuse adenomyosis

**DOI:** 10.1097/MD.0000000000033266

**Published:** 2023-03-31

**Authors:** Sang Ho Yoon, Grace J. Lee, Hye Jung Cho, Hayan Kwon, Bo Seong Yun, Chae Hyeong Lee, Hyun Soo Park, Ju-Won Roh

**Affiliations:** a Department of Obstetrics and Gynecology, Dongguk University Ilsan Hospital, Goyang, Republic of Korea; b Dartmouth College, Hanover, NH; c Department of Obstetrics and Gynecology, National Health Insurance Service Ilsan Hospital, Goyang, Republic of Korea; d Department of Obstetrics and Gynecology, Institute of Women’s Life Medical Science, Yonsei University College of Medicine, Seoul, Republic of Korea; e Department of Obstetrics and Gynecology, CHA Ilsan Medical Center, CHA University, Goyang, Republic of Korea.

**Keywords:** adenomyomectomy, adenomyosis, fertility preserving surgery, infertility

## Abstract

Beneficial and detrimental effect of surgical adenomyomectomy is still controversial in infertile women with severely diffuse adenomyosis. The primary objective of this study was to assess whether a novel method of fertility-preserving adenomyomectomy could improve pregnancy rates. The secondary objective was to evaluate whether it could improve dysmenorrhea and menorrhagia symptoms in infertile patients with severe adenomyosis. A prospective clinical trial was conducted between December 2007 and September 2016. Fifty women with infertility due to adenomyosis were enrolled in this study after clinical assessments by infertility experts. A novel method of fertility-preserving adenomyomectomy was performed on 45 of 50 patients. The procedure included T- or transverse H-incision of the uterine serosa followed by preparation of the serosal flap, excision of the adenomyotic tissue using argon laser under ultrasonographic monitoring, and a novel technique of suturing between the residual myometrium and serosal flap. After the adenomyomectomy, the changes in the amount of menstrual blood, relief of dysmenorrhea, pregnancy outcomes, clinical characteristics, and surgical features were recorded and analyzed. All patients obtained dysmenorrhea relief 6 months postoperatively (numeric rating scale [NRS]; 7.28 ± 2.30 vs 1.56 ± 1.30, *P* < .001). The amount of menstrual blood decreased significantly (140.44 ± 91.68 vs 66.33 ± 65.85 mL, *P* < .05). Of the 33 patients who attempted pregnancy postoperatively, 18 (54.5%) conceived either by natural means, in vitro fertilization and embryo transfer (IVF-ET), or thawing embryo transfer. Miscarriage occurred in 8 patients, while 10 (30.3%) had viable pregnancies. This novel method of adenomyomectomy resulted in improved pregnancy rates, as well as relief of dysmenorrhea and menorrhagia. This operation is effective in preserving fertility potential in infertile women with diffuse adenomyosis

## 1. Introduction

Adenomyosis is a pathological finding characterized by heterotopic growth of endometrial glands and stroma into the myometrium and is accompanied by a varying degree of muscular hypertrophic changes. Adenomyosis has been suggested to be associated with menstrual abnormalities, and affected women have compromised fertility; they often need assisted reproductive technology (ART) for reproduction.^[1,2]^

Like endometriosis, adenomyosis can impair successful pregnancy during ART, although the relation between uterine adenomyosis and infertility is not clearly defined. Adenomyosis can affect uterine or endometrial receptivity, and it leads to increased abortion rates.^[3,4]^ Adenomyosis is difficult to treat in infertile patients who need uterine conservation. Medical treatment is often transient, and hysterectomy cannot preserve the patient fertility, even though it has been a standard surgical treatment for adenomyosis.^[5]^ To date, there has been no consensus on the most appropriate therapeutic method for improving fertility outcomes in infertile patients with adenomyosis.

Several studies reported that various surgical methods, including endometrial resection, open or laparoscopic myometrial reduction by electrocautery, and excision of adenomyosis, reduced menorrhagia and dysmenorrhea as well as the need for hysterectomy in patients with adenomyosis.^[5–11]^ Furthermore, a few studies reported that patients were able to achieve pregnancy after adenomyomectomy in even diffuse adenomyosis, suggesting that the surgery is a conservative and effective treatment method for adenomyosis.^[12–16]^ Considering these roles of uterus-sparing surgery in relieving the symptoms of menstruation and preserving fertility, it can be postulated that maximal surgical reduction of adenomyotic tissue could be another treatment option for infertile patients with adenomyosis.

We modified previously reported conservative methods to facilitate effective reduction of diffuse adenomyotic lesions by including T- or transverse H-incision, utilization of an argon laser, sonographic measurement of the pathological lesion during the operation, and a novel suture technique for uterine reconstruction. We intended to improve menstrual symptoms, increase pregnancy rates, and decrease obstetrical complications after maximal reduction of pathologic adenomyotic tissue while preserving the endometrium and normal myometrial tissues. Uterus-sparing surgery appears to be an attractive option for treating adenomyosis and preserving fertility, but there is a paucity of well-designed prospective studies evaluating fertility and pregnancy outcomes after surgery.

The primary objective of this study was to assess whether this novel method of adenomyomectomy could improve pregnancy rates, and secondary goal was to evaluate whether it could improve dysmenorrhea and menorrhagia symptoms in infertile patients with severe adenomyosis via a well-designed prospective trial.

## 2. Materials and methods

### 2.1. Study participants

A prospective clinical trial was conducted between December 2007 and September 2016 at Dongguk University Ilsan Hospital, South Korea. Fifty study participants were selected based on the following criteria: patients diagnosed with diffuse adenomyosis via pelvic ultrasonography and magnetic resonance imaging (MRI); patients who had excessive menstrual flow (≥80 mL by pictogram) and severe dysmenorrhea (numeric rating scale [NRS] ≥ 6) that disrupted daily life; patients who had been diagnosed with primary or secondary infertility for more than a year, and their infertility was determined to be due to adenomyosis based on clinical assessment by an infertility expert; patients who did not have any other medical conditions, excluding iron deficiency anemia; patients who failed to conceive after treatment with drugs, such as gonadotropin-releasing hormone agonist (GnRHa); and infertile patients who had adenomyosis and other infertility factors, such as ovulation disorder and blocked fallopian tubes, yet their implantation failure was determined to be due to adenomyosis after more than 2 failed attempts to conceive through in vitro fertilization and embryo transfer (IVF-ET) and/or ovulation induction.

**Figure 1.**
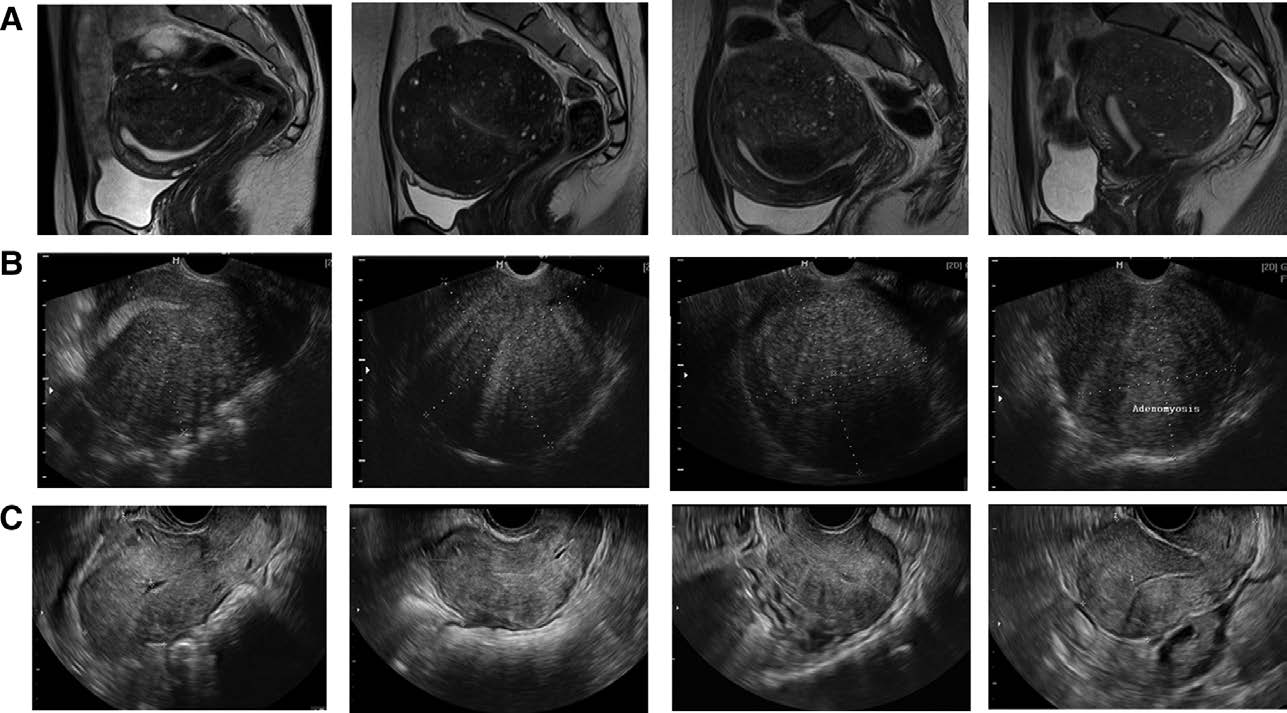
Representative images of MRI and trans-vaginal ultrasonography. (a) MRI scans of 4 different patients show adenomyosis. (b) Trans-vaginal ultrasonography was used to measure the adenomyotic lesion. (c) Trans-vaginal ultrasonography 3 to 6 mo follow-up after surgery. MRI = magnetic resonance imaging.

All patients underwent gynecological examinations and workup, including pelvic ultrasonography and MRI (Fig. 1), and were classified as having infertility due to adenomyosis. Adenomyosis was diagnosed was made upon identifying abnormalities such as the presence of globular shape uterus, asymmetrical thickening and hypoechogenic cystic feature of myometrium, fan-shaped posterior shadowing and irregular junctional zone.^[17,18]^ Dysmenorrhea was estimated by the highest number on the NRS during the last cycle of the menstrual period prior to diagnosis, and menorrhagia was identified by the amount of menstrual blood loss measured by the menstrual pictogram.^[19]^ “Severe adenomyosis” was defined as a case in which a patient exhibits a diffuse pattern of adenomyosis, experiences severe menstrual symptoms interfering with their daily life, and is infertile due to adenomyosis despite more than a year of pregnancy attempt via methods such as ART.

### 2.2. Ethical approval

The Institutional Review Board of Dongguk University Ilsan Hospital approved this study (IRB No. 2007-24). Written informed consent was obtained from all participants. All study procedures were performed in accordance with the guideline of Act on Bioethics and Sefety of South Korea. This study has been registered in Clinical Research Information Service, Republic of Korea (KCT0004135, July 11, 2019).

### 2.3. Adenomyomectomy procedure

**Figure 2.**
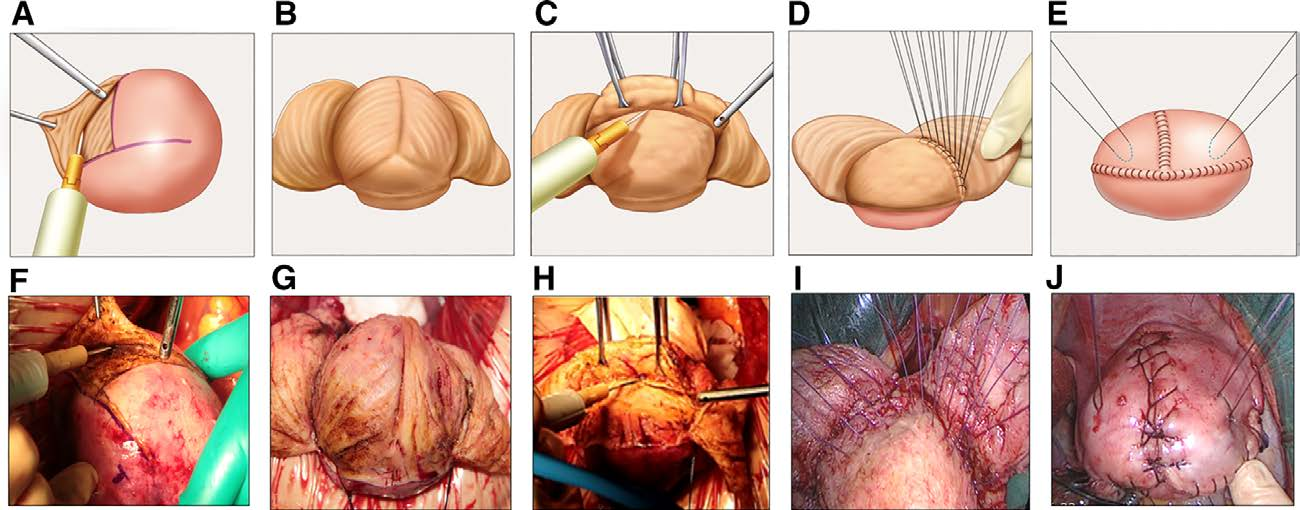
Schematic (a–e) and actual images (f–j) of the adenomyomectomy procedure. (a & f) A T-shaped incision is made on the uterine wall. (b & g) The serosal flap exposes adenomyotic tissue. (c & h) The adenomyotic tissue is shaved using the Argon laser. (d & i) The border between the serosal flap and residual myometrium is sutured. (e & j) The serosal flap is closed and fixed to the underlying myometrium via fixation suture.

We designed a novel operative procedure consisting of the following methods. First, an initial measurement of the adenomyotic lesion was made using ultrasonography and MRI (Fig. 1). Then, a conventional laparotomy was prepared. To identify the endometrial cavity, a balloon uterine catheter was inserted into the endometrial cavity. After entering the peritoneal cavity and confirming the location of the adenomyosis, a “T” or “transverse H” incision was made on the adenomyotic wall to peel and preserve the serosa for later uterine reconstruction (Fig. 2a and f). The T-shaped incision was made as if peeling off the entire outer layer of the uterus (Fig. 2b and g). A transverse H-shaped incision was made only in 2 cases where there was a large lesion in the isthmus.

The adenomyotic region was wide and lacked clear boundaries; hence, an argon laser (Valleylab^TM^ Force Argon II, Medtronic) with a flow rate of 4L/min, rather than a scalpel, was used to peel off the adenomyotic tissue in a direction parallel to the endometrial plane (Fig. 2c and h). The entire adenomyotic lesion was not removed at once; rather, small amounts of the adenomyotic tissue, 2 to 3 mm thick, were shaved off with the argon laser several times. Throughout this step, the thickness of the residual myometrium was frequently measured using intraoperative portable ultrasonography with abdominal probe (HM70A, SAMSUNG MEDISON, KOREA). When the thickness of the residual myometrium was confirmed to be approximately 1 cm, no further excisions were made, and uterus reconstruction was initiated, even though some adenomyosis appeared to remain.

As illustrated in Figure 2d and i, the absorbable sutures were carefully used along the border between the serosal flap and the residual myometrium to minimize dead space and hematoma formation. After the uterine serosa was closed, fixation suture (fixation of the serosal flap to the underlying myometrium to reduce the dead space) was applied (Fig. 2e and j). An adhesion-prevention barrier was applied on the uterine surface before closing the peritoneum.

After adenomyomectomy, the weight of the resected adenomyotic tissue, time of operation, amount of bleeding, and complications were recorded. Before and after the reductive operation, all patients received 3 cycles of GnRHa therapy, and their serum cancer antigen 125 (CA125) levels were measured. Symptom relief of menorrhagia and dysmenorrhea were evaluated by pictogram and by NRS, respectively. MRI and ultrasonography were used to confirm the remission of adenomyosis.

### 2.4. Antepartum management

After 6 months of adenomyomectomy, participants could try to conceive and the method of pregnancy was left to the patients’ preference, including natural means or ART.

In antepartum, to prevent preterm birth and uterine rupture during pregnancy, progestin treatment was provided to all patients for up to 34 weeks of gestation. They were administered vaginally once a day (Utrogestan, vaginal capsule 200 mg, Hanwha Pharma, Seoul, Korea) or injected intramuscularly every week (Progesterone Depot 250 mg, Jenapharm, Jena, Germany), depending on the patients’ preferences. A cesarean section was recommended as the delivery mode. Delivery timing was individualized, but we generally planned for birth at the early term in accordance with the recommendations set by the American College of Obstetricians and Gynecologists.^[20]^ Pregnancy outcomes including clinical pregnancy, miscarriage, viable pregnancy and live births were evaluated.

### 2.5. Statistical analysis

The data are presented as mean ± SD or median with ranges. Comparison of clinical outcomes before and after surgery was performed using the paired *t* test. All data analyses were performed using the Statistical Package for the Social Sciences software (version 21.0, IBM SPSS Inc, Armonk, NY). Two-sided *P* values < .05 were considered statistically significant.

## 3. Results

### 3.1. Clinical and surgical outcomes

The clinical characteristics and surgical features of the patients are summarized in Table 1. The mean age and the duration of infertility were 35.60 ± 3.33 years and 55.48 ± 48.24 months, respectively. The mean weight of the excised specimens of adenomyosis was 94.15 ± 56.63 g. Pelvic endometriosis existed in 17 (34%) patients, and postoperative complications, including uterine shrinkage, premature ovarian insufficiency, ureter fistula, and subfascial hematoma, occurred in 4 patients. “Uterine shrinkage” refers to a decrease in the length of the uterus to <4 cm after surgery, accompanied by the absence of menstruation despite normal ovarian function.

**Table 1:** Clinical and surgical features of enrolled patients.

Characteristics (n = 50)	
Age (yr)	35.60 ± 3.33
Etiology of infertility	
Unexplained with adenomyosis	42 (84.0)
Ovulation factor with adenomyosis	5 (10.0)
Male factor with adenomyosis	3 (6.0)
Duration of infertility (mo)	55.48 ± 48.24
Nullipara	46 (92)
Surgical features (n = 45)	
Operation time (min)	321.02 ± 97.06
Volume of excised adenomyosis (g)	94.15 ± 56.63
Estimated blood loss (mL)^a^	507.00 ± 404.95
Pelvic endometriosis	17 (37.8)
Postoperative complications	4 (8.9)
Pelvic infection with ureter fistula	1
POI immediately after the operation	1
Subfascial hematoma	1
Shrinkage of uterus	1

Values are presented as mean ± SD, n (%) and median (range).

POI = premature ovarian insufficiency.

Six months postoperatively, all patients obtained symptom relief of dysmenorrhea (NRS; 7.28 ± 2.30 vs 1.56 ± 1.30, *P* < .001). The amount of menstrual blood (140.44 ± 91.68 vs 66.33 ± 65.85 mL, *P* < .05) and CA125 levels (187.75 ± 221.13 vs 20.78 ± 19.28 IU/mL, *P* < .05) decreased significantly (Table 2).

**Table 2:** Clinical outcomes of adenomyomectomy in infertile patients.

	Preop	Postop 6 mo	*P* value
CA125 (IU/mL)	126.30 (9.95–885.90)	16.54 (5.40–58.60)	.002*
Dysmenorrhea‡	7.28 ± 2.30	1.56 ± 1.30	<.001†
Menorrhagia (mL)§	140.44 ± 91.68	66.33 ± 65.85	.009†

Values are presented as median (range) and mean ± SD.

CA125 = cancer antigen 125.

* Evaluated by the Wilcoxon signed rank test.

† Evaluated by the paired *t* test

‡ Evaluated by Numeric Rating Score (NRS) score.

§ Evaluated by pictogram.

### 3.2. Pregnancy outcomes

**Figure 3.**
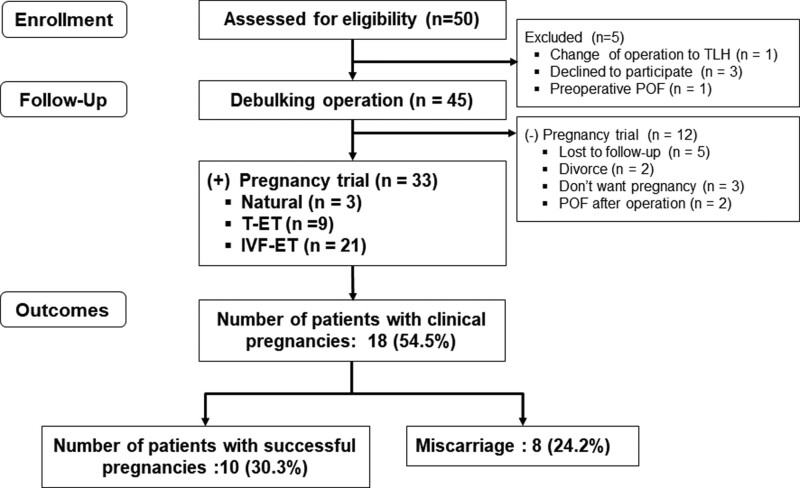
Flowchart reporting the pregnancy outcome of study subjects after adenomyomectomy.

Pregnancy outcomes are shown in Table 3. Fifty patients were enrolled in this study, 45 of whom underwent surgery. Postoperatively, 33 patients attempted pregnancy. Five patients were lost to follow-up. Eighteen of 33 patients (54.5%) who attempted pregnancy conceived either by natural means, IVF-ET, or thawing ET postoperatively. Spontaneous abortion occurred in 5 patients, and ectopic pregnancy occurred in 3 patients (Fig. 3). The viable pregnancy rate was 30.3% (10/33); 3 patients delivered at preterm (31 + 2, 36 + 2, and 36 + 3 weeks), and 8 patients delivered at full-term (median gestational age at birth was 37 + 2 weeks, range: 31 + 2 ~ 37 + 6 weeks). The cause of birth at 31 + 2 weeks of gestation was preterm labor and preterm premature rupture of membranes. The other 2 cases were elective cesarean deliveries at 36 + 2 and 36 + 3 weeks of gestation. All patients underwent cesarean section. The number of live births was 13, including 2 cases of twin pregnancies and 1 patient who successfully gave birth twice. There were no cases of uterine rupture or cesarean hysterectomy during the peripartum period.

**Table 3:** Pregnancy outcomes in infertile patients following adenomyomectomy.

No. of patients who tried to be pregnant (n = 33)	
Failure	15 (45.5)
Clinical pregnancies	18 (54.5)
Miscarriage	8 (24.2)
Spontaneous abortion	5
Ectopic pregnancy	3
Viable pregnancies	10 (30.3)
Singleton	8
Twin	2
Term	8
Preterm	3
Live births	13*

Data are n (%).

* Two cases of twin pregnancies and 1 patient who successfully gave birth twice.

## 4. Discussion

The present study evaluated the clinical efficacy of conservative debulking surgery in infertile patients with severely diffuse adenomyosis. Our novel method of fertility-preserving adenomyomectomy resulted in increased pregnancy rates with successful outcomes, as well as symptom relief of dysmenorrhea and menorrhagia. These results suggested that this method of reductive operation could be effective in preserving fertility in infertile women with adenomyosis. To the best of our knowledge, this is one of the few reports on the clinical pregnancy outcomes of uterus-sparing surgery in diffuse adenomyosis through a prospective clinical trial.

Although several studies have been conducted to determine the efficacy of adenomyosis reductive operation, many outcomes have been limited to the relief of menstrual symptoms. The relationship between adenomyosis reductive operation and fertility and pregnancy outcomes in diffuse adenomyosis remains poorly understood.^[6,21,22]^ Recently, there were a few prospective trials that yielded variable pregnancy rates after excisions of adenomyosis: 14.3% (2/14), 61.5% (16/26), 2.3% (2/86), 30% (21/70), and 40.8% (56/137).^[12–16]^ In systemic review of 18 studies (retrospective and prospective) and 1396 infertile women with focal and diffuse adenomyosis, pregnancy rate after fertility-sparing surgery for diffuse-type adenomyosis was 34.1%.^[23]^

In our cohort, 54.5% (18/33) of patients conceived postoperatively either by natural means, IVF-ET, or thawing ET. After reduction surgery, all patients were recommended to try to conceive through ART. However, some patients refused ART procedures or became pregnant naturally. Our result, which was relatively higher than those of previous studies, is remarkable considering the strict criteria used to select patients with severe adenomyosis. All participants enrolled in this study consisted of infertile patients who failed to conceive via IVF-ET. Although it is difficult to compare the results directly, in a retrospective study included 241 IVF cycles of women with adenomyosis, the pregnancy rate was low at 25.2% to 39.5% without surgery.^[24]^ More importantly, there were no serious peripartum complications in our study, such as uterine rupture and postpartum hemorrhage. In systemic review, uterine rupture was reported in 6.8% of pregnant women with diffuse adenomyosis after fertility-sparing surgery.^[23]^

The proportion of patients who attempted pregnancy was different among studies, accounting for the widely variable pregnancy rates.^[12–15]^ In addition, different surgical methods could have resulted in different pregnancy rates. We made a T-incision on the uterine wall and created a serosal flap for broad exposure and complete reconstruction. The previously reported transverse H-shaped incision^[6]^ was used for only 2 cases with large lesions in the isthmus. This serosal flap provided sufficient exposure of the adenomyotic tissue, which is instrumental to maximal removal of the lesion. The novel surgical methods introduced in this study have certain distinct features. First, we used an argon laser for the resection of adenomyotic tissue. The argon laser is sharper than the usual metal scalpel, and its cutting power is so high that it is also called a “laser scalpel.” The argon laser also possesses a selective coagulating ability for red blood cells because its wavelength is absorbed by hemoglobin. Additionally, it has an advantage over other electrical devices in that it minimizes damage to the surrounding tissue. By using the argon laser, adenomyosis resection can be performed more delicately, and the surgical field can be maintained more clearly throughout the operation. Second, we used portable intraoperative ultrasonography to monitor the resection of adenomyosis and residual myometrial thickness from the endometrium throughout the surgery. Although there was a trial in which the uterine endometrial cavity was opened for removal of adenomyotic tissue,^[14]^ we believe such a method could be detrimental to the preservation of the endometrium and fertility with successful outcomes. Moreover, we believe that keeping the myometrium at least 1 cm thick is particularly important to prevent uterine rupture during future pregnancy. Inserting a balloon catheter, such as a foley catheter or uterine manipulator, into the endometrium can help identify the endometrium. Finally, a new surgical technique for myometrial repair was introduced in this study. More specifically, the boundary between the myometrial lateral border and the serosal flap, a region with a high risk of bleeding, was sutured in a careful manner, preventing malunion of the uterine wall and intramyometrial defect. Also, the novel technique of occasional fixation suture, implemented to prevent hematoma or seroma occurrence between the residual myometrium and serosal flap, was helpful in minimizing complications.

Although adenomyomectomy primarily aims to increase pregnancy success rates, relieving dysmenorrhea and menorrhagia is also crucial because if symptoms persist, many patients are forced to abandon their active pursuit of pregnancy. Pelvic pain, especially dysmenorrhea, can also be caused by other diseases accompanying adenomyosis, especially endometriosis, which are described as different expressions of the same pathological process.^[25]^ A positive correlation between the severity of dysmenorrhea and the occurrence of endometriosis, especially deep infiltrative endometriosis, has been reported.^[26]^ In this study, about 38% of patients underwent surgery for both adenomyosis and endometriosis at the same time, which is consistent with this finding (Table 1). The mechanism behind how endometriosis and adenomyosis cause dysmenorrhea is not clear. A few studies found that pelvic pain may be due to the inflammatory response of multiple leucocytes in the endometriotic plaque and the nerve fiber present in the eutopic endometrium.^[27,28]^ In this study, adenomyomectomy was found to be highly effective in relieving dysmenorrhea in patients with or without endometriosis (Table 2).

There were 4 cases of postoperative complications in the beginning of the study: subfascial hematoma, ureter fistula, shrinkage of the uterus, and premature ovarian insufficiency (Table 1). Of the 4 cases, all complications excluding subfascial hematoma occurred in patients with pelvic endometriosis. Adhesions in the pelvis resulting from endometriosis and previous surgeries may impact postoperative complications. Ovarian insufficiency, rather than due to adenomyomectomy itself, is thought to be due to the decrease in periovarian blood supply and ovarian reservoir that might result from extensive adhesiolysis and periorvarian adhesiolysis performed in patients with pelvic endometriosis and adhesion. Ureter fistula also occurred in patients with severe pelvic endometriosis who had pelvic infection after extensive adhesiolysis.

To minimize these complications, meticulous and careful procedures are essential. In addition, pre- and postoperative GnRHa treatments may be helpful in improving surgical outcomes by minimizing the endometriosis-induced inflammatory reaction and reversible adenomyotic lesion prior to the operation. These treatment methods also aid in uterine muscular healing postoperatively by blocking menstrual blood from integrating into the intramuscular collection. To avoid postoperative ovarian insufficiency, preoperative cryopreservation of the embryo can be considered in high-risk patients with ovarian insufficiency.

Although this study was conducted before the Morphological Uterus Sonographic Assessment consensus was published, the adenomyosis diagnostic criteria used in this were not significantly different from those of Morphological Uterus Sonographic Assessment consensus.^[29]^ The criteria used to determine “severe” adenomyosis in this study were not based on current imaging definitions either. However, upon reclassification using recently reported imaging classifications of adenomyosis, all but 3 patients were “diffuse, severe adenomyosis.”^[30]^

CA125 screening, though currently not typically recommended for routine follow-up, is instrumental in fully assessing the efficacy of adenomyosis treatment and evaluating the recurrence of adenomyosis. High CA125 levels commonly present in individuals with adenomyosis, and numerous studies show that CA125 levels positively correlate with development and severity of adenomyosis. Recent research has also established a positive correlation between CA125 levels and the amount of adenomyosis remaining postadenomyomectomy and demonstrated that a reduction in CA125 levels were evident in successful treatments of adenomyosis.^[31,32]^ Our study also supports these findings: there was a significant decrease in CA125 levels following adenomyosis excision (Table 2). These results highlight the clinical value of CA125 screening in determining the treatment extent of adenomyosis elimination and measuring postoperative, remnant adenomyosis. Adenomyosis, endometriosis and gynecological cancer such as endometrial cancer are often predicted to have a common pathogenetic mechanism, and they are often found to occur together because they have a common genetic and hormonal factor. Some studies have even claimed that these conditions are related, but they have not shown consistent results nor reached an accurate conclusion yet.^[33,34]^

The association between adenomyosis and other diseases still requires extensive clinical and molecular investigation. However, in any situation, the presence of adenomyosis does not exclude the possibility of comorbid diseases, so if there are symptoms such as abnormal uterine bleeding, efforts should be made to initially exclude adenomyosis as the primary cause.

We found it most difficult to select patients who fit this study criteria due to the controversial nature of conducting clinical research. Finding the right timing for surgery will be an essential research task in the future in order to prevent delayed surgeries that result in infertility and premature surgeries, resulting from hasty decision making, that are ultimately harmful and/or unnecessary.

The limitation of this study was the relatively small sample size and not the comparative study without surgery; hence, future studies with more patients will be helpful in evaluating the efficacy of this surgical method. Therefore, we are planning a randomized controlled trial comparing the reproductive and pregnancy outcomes between women who have conducted this novel adenomyomectomy and those who have not in infertile women with diffuse adenomyosis.

In conclusion, this study reports successful pregnancy rates and outcomes after reduction surgery. These findings imply that the novel method of adenomyomectomy as a uterus-sparing surgery can alleviate the symptoms of dysmenorrhea and menorrhagia, improve pregnancy rates, and conserve fertility potential in infertile women with severely diffuse adenomyosis.

## Acknowledgments

Jimin J. are greatly acknowledged for helping the preparation of the manuscript.

## Author contributions

**Conceptualization:** Ju-Won Roh, Sang Ho Yoon.

**Data curation:** Grace J. Lee, Hye Jung Cho, Hayan Kwon, Chae Hyeong Lee, Hyun Soo Park.

**Formal analysis:** Ju-Won Roh, Sang Ho Yoon, Bo Seong Yun.

**Supervision:** Ju-Won Roh.

**Writing – original draft:** Ju-Won Roh, Sang Ho Yoon, Grace J. Lee.

**Writing – review & editing:** Ju-Won Roh, Sang Ho Yoon, Grace J. Lee, Hye Jung Cho, Hayan Kwon, Bo Seong Yun, Chae Hyeong Lee, Hyun Soo Park.
